# The Metabolic Changes between Monolayer (2D) and Three-Dimensional (3D) Culture Conditions in Human Mesenchymal Stem/Stromal Cells Derived from Adipose Tissue

**DOI:** 10.3390/cells12010178

**Published:** 2023-01-01

**Authors:** Paulina Rybkowska, Klaudia Radoszkiewicz, Maria Kawalec, Dorota Dymkowska, Barbara Zabłocka, Krzysztof Zabłocki, Anna Sarnowska

**Affiliations:** 1 Translational Platform for Regenerative Medicine, Mossakowski Medical Research Institute, Polish Academy of Sciences, 02-106 Warsaw, Poland; 2 Molecular Biology Unit, Mossakowski Medical Research Institute, Polish Academy of Sciences, 02-106 Warsaw, Poland; 3 Laboratory of Cellular Metabolism, Nencki Institute of Experimental Biology, Polish Academy of Science, 02-093 Warsaw, Poland

**Keywords:** MSC, ASC, DFAT, 3D culture, three-dimensional culture, spheroid, spheres, metabolism, mitochondria, OXPHOS, glycolysis, ATP, hypoxia

## Abstract

Introduction: One of the key factors that may influence the therapeutic potential of mesenchymal stem/stromal cells (MSCs) is their metabolism. The switch between mitochondrial respiration and glycolysis can be affected by many factors, including the oxygen concentration and the spatial form of culture. This study compared the metabolic features of adipose-derived mesenchymal stem/stromal cells (ASCs) and dedifferentiated fat cells (DFATs) cultivated as monolayer or spheroid culture under 5% O_2_ concentration (physiological normoxia) and their impact on MSCs therapeutic abilities. Results: We observed that the cells cultured as spheroids had a slightly lower viability and a reduced proliferation rate but a higher expression of the stemness-related transcriptional factors compared to the cells cultured in monolayer. The three-dimensional culture form increased mtDNA content, oxygen consumption rate (OCR) and extracellular acidification rate (ECAR), especially in DFATs-3D population. The DFATs spheroids also demonstrated increased levels of Complex V proteins and higher rates of ATP production. Moreover, increased reactive oxygen species and lower intracellular lactic acid levels were also found in 3D culture. Conclusion: Our results may suggest that metabolic reconfiguration accompanies the transition from 2D to 3D culture and the processes of both mitochondrial respiration and glycolysis become more active. Intensified metabolism might be associated with the increased demand for energy, which is needed to maintain the expression of pluripotency genes and stemness state.

## 1. Introduction

The therapeutic efficacy of mesenchymal stem/stromal cells (MSCs) application is based mostly on their adjuvant properties although possible differentiation toward the desired phenotype might be taken into consideration. The adjuvant properties are mostly associated with MSCs proliferation, clonogenic potential, viability, and differentiation, which results, among others, from culture conditions. Currently, to increase cell proliferation and viability, different biomaterials and scaffolds are being tested [1,2,3]. Despite the development of the engineering methods, we are still unable to maintain stem/stromal cells at their therapeutically best and most beneficial stage, which seems crucial for the development of this field of transplantology. 

The time length of the culture is one of the limiting factors, as a growing number of passages causes senesce and a reduction of stem cells’ valuable properties [4].

The therapeutic potential of MSCs may also be affected by limited and imperfect cell-to-cell contact in the monolayer culture and a high, artificial oxygen concentration (21%) in the culture environment. It has been confirmed that in their natural niche MSCs adhere tightly to each other and are in close contact. They constitute a heterogeneous population and cooperate with other cell types, such as hematopoietic cells, precursor cells, or already differentiated and mature cells. This close cooperation is needed for the free exchange of signals and impulses that enables differentiation when needed [5]. To mimic the natural conditions present in the natural stem cell niche, specific environmental conditions/supplements are used to stimulate MSCs to form the three-dimensional structures: aggregates or spheroids. There are various methods to perform three-dimensional cell arrangement, the most popular of which seem to be the following: culture on the low attachment surface plates [6], hanging drop, spinner culture, or magnetic structuring [7]. It has been shown that the spatial forms of mesenchymal cells’ culture support the maintenance of therapeutically desirable properties of these cells in comparison to the cells cultured as a monolayer, in both in vitro and in vivo experiments [8]. Moreover, MSCs spheroids have demonstrated many therapeutic advantages, such as higher pluripotency gene expression [9,10], early neural and oligodendrocyte markers expression [11], higher cytokine and chemokine secretion [12,13] enhanced osteogenic, adipogenic and neurogenic differentiation [14], [6] higher secretion of anti-inflammatory and proangiogenic factors [15] or increased exosome release [16,17]. In the animal models, it was further shown that three-dimensional MSC forms promoted vascularization [18,19], supported angiogenesis [20], bone [21], and cartilage regeneration [22], and might exert a positive therapeutic effect on colitis [23], ischemic kidney injury [24] and brain infarct [25,26]. However, the precise molecular mechanisms conditioning the therapeutic properties of the MSC spheroids are not yet entirely known.

Oxygen concentration also has a very significant impact on the cell therapeutic properties, both adjuvant and differentiation potential. In the natural niche, the oxygen concentration varies depending on the tissue and ranges from 1–7% O_2_ [27]. Based on oxygen availability, the MSC cells can rely on glycolysis and/or oxidative phosphorylation (OXPHOS) to maintain cell homeostasis. The appropriate balance between these two mechanisms is needed to provide a sufficient amount of energy in the form of ATP and to regulate the release of Reactive Oxygen Species [28,29]. Therefore, it seems crucial to provide MSCs with adequately low oxygen, close to the physiological concentration in their environment, to support the maintenance of stemness. The effects of the reduction of oxygen content in the culture from commonly used 21% O_2_ (atmospheric O_2_) to lower values, e.g., 5% O_2_ (physiological normoxia/physioxia) have been extensively studied in terms of the therapeutic abilities of the MSCs. It is known that physiological normoxia conditions have a beneficial effect on cell viability, proliferation, colony-forming ability, migration, paracrine abilities, and the expression of the stemness markers [30,31,32]. As further shown, in reduced oxygen conditions cell metabolism may reconfigure and switch between oxidative phosphorylation and glycolysis, but this reconfiguration probably depends on the stage of cell development [33]. For instance, cancer cells still rely on anaerobic respiration [34] despite the presence of oxygen in the environment, and this phenomenon is known as the Warburg effect. Similarly, embryonic stem cells (ESCs) or induced pluripotent stem cells (iPS) have been characterized to preferentially rely on anaerobic glycolysis [35,36,37,38]. According to previous research [31,32,33], physioxic conditions (5% O_2_) were found to be beneficial for MSC proliferation and vital potential.

**Figure 1:**
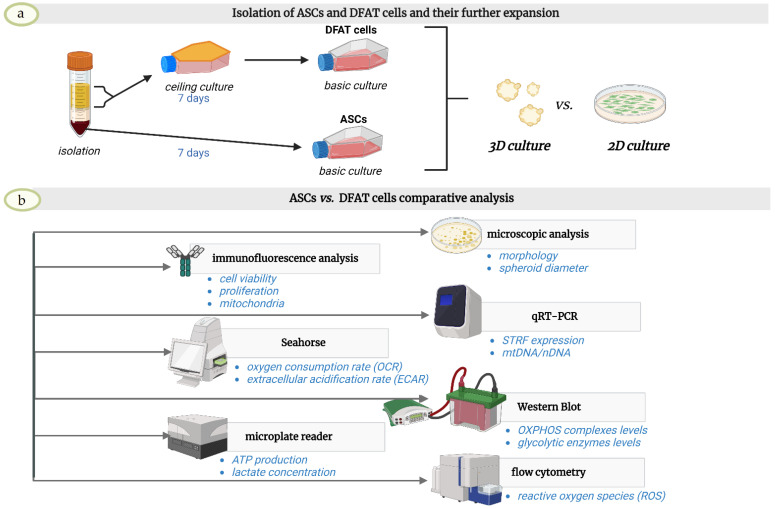
Overview of experimental steps. (**a**) Isolation of ASCs and DFAT cells and their further expansion (**b**) ASCs and DFAT cells comparative analysis.

In this study, we analyzed the combined effect of spatial form culture and physioxic conditions, resembling the natural cell niche, on metabolic reconfiguration and the effects that it produced on cell viability, proliferation, and stemness (Figure 1). We mainly focused on oxidative mitochondrial respiration and anaerobic glycolysis activity and their impact on the therapeutic abilities of these cells. For our experiments, we used two cell populations derived from adipose tissue: standard Adipose-Derived Stem/ Stromal Cells (ASCs) and Dedifferentiated Fat Cells (DFATs), which have recently been shown to be a more homogenous population with favorable properties, such as enhanced stemness gene expression, better neurogenic and neuroprotective potential [39], higher neovascularization, angiogenic and neurorestorative potential due to increased factors secretion [40,41]. 

Our hypothesis was that the three-dimensional culture of ASCs and DFATs in physiological oxygen concentrations might influence the reconfiguration of cell metabolism. Such a change may lead to potentially better therapeutic properties of the three-dimensional cells when compared to the adherent. Moreover, the novelty of this study is related to the use of DFAT cells, whose metabolism and spheroid culture have not been previously described.

## 2. Materials and Methods

### 2.1. Cell Isolation and Culture

Subcutaneous abdominal adipose tissue was acquired by the liposuction procedure from 7 healthy adult donors (4 females, 3 males, 30 ± 67 years old ± SD) from the Department of Plastic Surgery at Orlowski’s Clinical Hospital in Warsaw according to the Bioethical Committee at the Centre of Postgraduate Medical Education (No. 62/PB/2016) guidelines. All patients signed informed consent for participation in the study. The collected fat tissue was rinsed with PBS (Sigma-Aldrich, Saint Louis, MO, USA) with the addition of Penicillin-Streptomycin-Amphotericin B solution (1%, Gibco, ThermoFisher Scientific, Waltham, MA, USA) in 50 mL Falcon tubes (Beckton Dickinson, Franklin Lakes, NJ, USA) and digested by type VI GMP Grade Collagenase (Serva, Heidelberg, Germany). The adipose tissue was separated into three layers and ASCs and DFATs were collected. Both cells’ populations were seeded on 25 cm^2^ flasks (Nunc, ThermoFisher Scientific, Waltham, MA, USA) at the concentration of 2500 cells/cm^2^/5ml in a basic growth medium: MEM-α (Gibco, ThermoFisher Scientific), PLT Gold-Human Platelet Lysate (5%, Genos, Lodz, Poland) and Penicillin-Streptomycin-Amphotericin B solution (1%, Gibco, ThermoFisher Scientific) under 5% O_2_, 5% CO_2_ and 37 °C conditions. ASCs and DFATs were cultured until 70–80% of subconfluency and detached from the dishes using Accutase Cell Detachment Solution (Beckton Dickinson, Franklin Lakes, NJ, USA). 

### 2.2. Mesodermal Lineage Differentiation

For mesodermal lineage differentiation-osteogenesis, adipogenesis, and chondrogenesis-ASCs and DFATs were seeded on 24-well plates (Nunc, Thermo Fischer Scientific) at a density of 2500/cm^2^. For each lineage, cells were cultured in specific commercial differentiation media (Gibco, Thermo Fisher Scientific, Waltham, MA, USA). After 14 days of culture in adipogenic medium and chondrogenic medium, and after 21 days of osteogenic differentiation ASCs and DFAT were fixed in 4% PFA and stained: for adipogenesis: Oil Red (0.5%), for osteogenesis: Alizarin Red S (2%), chondrogenesis Alcain Blue (1%). The photographs were analyzed with Axio Vert.A1 (Carl Zeiss, Oberkochen, Germany) inverted microscope and ZEN software (Carl Zeiss, Oberkochen, Germany).

### 2.3. Flow Cytometry

The expression of specific mesenchymal surface markers was analyzed with Human MSC Analysis Kit (Beckton Dickinson), according to the producers’ protocol. Firstly, ASC and DFAT cells in passage 2 were detached from plates by Accutase Cell Detachment Solution (Beckton Dickinson) and suspended in BD Stain Buffer (Beckton Dickinson). The specific antibodies against CD90, CD73, CD105 (positive markers) and CD34, CD11b, CD19, CD45, and HLA-DR (negative markers) were added, and the cells were incubated in the dark at room temperature (RT) for 30 min. After incubation, the cells were washed, centrifuged, and suspended in Stain Buffer (Beckton Dickinson) and then analyzed by flow cytometry FASC Canto II. The obtained results were analyzed by using FlowJo software version 10.7.1 (Becton Dickinson, New Franklin Lakes, NJ, USA). 

### 2.4. ASC and DFAT Spheroid Formation

The 2nd passage cells were used to conduct the 3D spheroids cultures using anti-adhesive plates (Nunclon Sphera, ThermoFisher Scientific) at the density of 0.1 × 10^6^/mL in 5% O_2_, 5% CO_2_, and 37 °C conditions. The 3D spheroids culture medium for ASCs and DFATs was additionally supplemented with the FGF (20 ng/mL; PeproTech, London, UK) and EGF (20 ng/mL; PeproTech, London, UK), and heparin (2 µg/mL Sigma-Aldrich). The 3D spheroids were cultured for up to 72 h and then used for experiments.

### 2.5. Life-Dead Staining 

2D cultures and 3D spheroids of ASCs and DFATs were simultaneously stained with the green Calcein AM (Cal-AM, 0.25 µL/mL) dye and red ethidium homodimer-1 (1 µL/mL, EthD-1) dye (Invitrogen). The cells were incubated with a mix of dyes for 30 min in darkness at room temperature and then observed and photographed under fluorescence microscopy Axio Vert.A1 (Carl Zeiss, Oberkochen, Germany). To obtain the precise number of living and dead cells, the 3D spheroids were firstly disrupted with Accutase Cell Detachment (Beckton Dickinson) for 15 min, then stained with Cal-AM + EthD-1 and Hoechst 33342 dye (1 µg/mL, Sigma-Aldrich) for the nuclei staining. Live and dead ASCs and DFATs in both 2D and 3D forms were counted with the ZEN 2 Blue Edition software (Carl Zeiss, Oberkochen, Germany), according to the previously described protocols [11]. 

### 2.6. Immunofluorescence Staining and Imaging of Ki67 Expression

The Ki67 expression was analyzed with the use of the immunocytochemistry method, as previously described [39,42]. Firstly, ASC-2D and DFAT-2D cells were seeded on 24-well plates (ThermoFisher Scientific) at a concentration of 2500/cm^2^ and ASC-3D and DFAT-3D on 24-well anti-adhesive plates (ThermoFisher Scientific) at concentration of 0.1 ×10^6^/mL. In case of ICC staining of monolayer, ASC-2D and DFAT-2D cells were washed in PBS (ThermoFisher Scientific) and fixed in 4% PFA (Sigma-Aldrich) for 15 min at RT. Then, the samples were permeabilized and blocked with 0.2% Triton X-100 (Sigma Aldrich) in 1% Bovine Serum Albumin (Sigma-Aldrich) in 10% Goat Serum (Gibco) in PBS solution (ThermoFisher Scientific). In the next step, the cells were incubated with primary antibodies: anti-Ki67 (Sigma-Aldrich) and anti-Vimentin (Dako) for 24 h at 4 °C (Table 1). Then, the cells were washed twice with PBS (ThermoFisher Scientific) and incubated for 2 h with the secondary antibodies at RT (Table 2). Cells were mounted on slides and their nuclei were stained by Fluoromount-G with DAPI (ThermoFisher Scientific). Images of cells were taken with a confocal microscope Zeiss LSM780 (Carl Zeiss, Oberkochen, Germany) and Ki67 positive cells were counted with the use of ZEN 2 Blue Edition software (Carl Zeiss) and presented as a percentage of positive cells in relation to all cells. The immunocytochemistry procedures for ASC-3D and DFAT-3D, was performed according to the previously described method [43]. Spheroids were divided into two groups: one was dissociated using Accutase Cell Detachment Solution (Beckton Dickinson) and seeded back to the monolayer (2D). After 3 h of incubation, spheroid-derived ASCs and DFATs were stained according to the protocol for 2D culture presented above. The cell counting was made in ZEN 2 Blue Edition software (Carl Zeiss). The second group of 3D-ASC and 3D-DFAT cells (the spheroids) were fixed with 4% PFA (Sigma-Aldrich) for 20 min, permeabilized and blocked using 0.2% Triton X-100 (Sigma-Aldrich) and 6% Blocker™ Bovine Serum Albumin (ThermoFisher Scientific) in PBS solution (ThermoFisher Scientific) for 24 h, at RT. Next, the spheroids were washed in PBS (ThermoFisher Scientific) and stained using the procedure for 3D culture. In brief, the spheroids were incubated with primary antibodies: anti-Ki67 (Sigma Aldrich) and anti-Vimentin (Dako) for 24 h at 4 °C (Table 1). Then, the cells were washed again with PBS (ThermoFisher Scientific) and incubated with secondary antibodies for 24 h, at RT (Table 2). ASC-3D and DFAT-3D were mounted, and the nuclei were visualized with Fluoromount-G with DAPI (ThermoFisher Scientific) and photographs were taken with a confocal microscope Zeiss LSM780 (Carl Zeiss).

### 2.7. Quantitative RT-PCR Analysis 

Total RNA was isolated from 2D and 3D spheroids cell cultures using Total RNA Mini Plus Concentrator (A&A Biotechnology, Gdansk, Poland), according to the manufacturer’s protocol. RNA was eluted with 15 µL of RNase-free H_2_O (Sigma-Aldrich). The purity and RNA concentration was measured by NanoDrop ND-1000 (ThermoFisher Scientific). The complementary cDNA strand was obtained after reverse transcription, performed with the use of a High-Capacity RNA-to-cDNA Kit (Applied Biosystems, ThermoFisher Scientific), according to the manufacturer’s protocol. Following the reverse transcription, cDNA samples were diluted in RNase-free H_2_O (Sigma-Aldrich) and stored at −20 °C until they were further used. Quantitative RT-PCR reactions were performed using 3-color RT HS-PCR SYBR Green Master Mix (Applied Biosystems), primers: *NANOG, SOX2, OCT3/4, REX1, PFKFB3, G6PD, LDHA, PDHA, PFKM* (Table 3) and Applied Biosystems^®^ 7500 Real-Time PCR System (Applied Biosystems). The relative gene expressions were calculated using the 2^−ΔΔCt^ method with *ACTB* as a reference gene according to the previously described method [44]. 

### 2.8. Mitochondrial Content 

The mitochondrial content was estimated by fluorescence of MitoTracker™ Green FM (M7514, ThermoFisher Scientific, Waltham, MA, USA). The ASC-2D and DFAT-2D cells were seeded on a monolayer at a concentration of 2500 cells/cm^2^. In the case of ASC-3D and DFAT-3D spheroids, the cells were seeded in the concentration of 0.1 × 10^6^ /mL on a 24-well plate (ThermoFisher Scientific) and cultured for 3 days, then reseeded back to the monolayer. In the next step, all variants of ASCs and DFATs were incubated with 100 nM MitoTracker Green dye for 30 min at 37 °C in the dark. The cells were then washed twice with PBS (ThermoFisher Scientific), and the fluorescence ratio was measured parallelly by FLUOstar OMEGA spectrophotometer (BMG LabTech), according to previously described protocols. Immunocytochemical staining was performed to visualize mitochondrial shape and arrangement. Both spheroid and monolayer ASCs and DFATs were stained using previously described methods (2.3), but with the use of primary antibodies: anti-Fibronectin (Sigma-Aldrich) and anti-Mitochondrial surface (Merck) (Table 1) and secondary antibodies (Table 2). The photographs of the cells were taken with a confocal microscope Zeiss LSM780 (Carl Zeiss).

### 2.9. mtDNA/nDNA Ratio

Relative mtDNA copy numbers were estimated by real-time quantitative PCR according to previously described methods [44,45]. Total DNA was isolated from ASCs and DFATs adherent and spheroid cultures by Genomic Mini Kit (A&A Biotechnology) according to the producer’s instructions. DNA probes were diluted in RNase-free H_2_O (Sigma-Aldrich), to equal concentration and stored at −20 °C until further use. 

The mtDNA content analysis was performed with TaqMan Master Mix (Applied Biosystems) on Applied Biosystems^®^ 7500 Real-Time PCR System (Life Technologies) using the following primers: MT16520F, MT35R, and MT16557TM probe. (5′-CATAAAGCCTAAATAGCCCACACG-3′), MT35R (5′- CCGTGAGTGGTTAATAGGGTGATA-3′) and MT16557TM probe (5′-FAM-AGACATCACGATGGATCACAGGTCT-TAMRA-3′). Nuclear DNA was measured by Kir4.1 amplification with KIR835F, KIR903R primers, and KIR857TM probe (5′-CCTTCCTTGGTTTGGTGGG-3′), KIR903R (5′- GCGCAAAAGCCTCCTCATT-3′) primers, and KIR857TM probe (5′-FAM-TGCCAGGTGACAGGAAAACTGCTTCAG-TAMRA-3′). The results were calculated as mtDNA/nDNA content based on the ΔΔCt method according to the previously described method [46].

### 2.10. Metabolic Phenotype

For the oxygen consumption rate (OCR) and extracellular acidification rate (ECAR) measurements, ASCs-2D and DFATs-2D were seeded at a density of 1 × 10^5^ cells/well and ASCs-3D and DFATs-3D at the concentration of 0.1 × 10^6^ cells/ mL in 8-well Seahorse XF HS Mini plates in MEMα (Gibco) containing 5% of PLT Gold-Human Platelet Lysate (Genos). After 3 days of culture, on the day of the test, the culture medium was exchanged for Agilent Seahorse XF Base Medium (Seahorse Bioscience, Billerica, MA, USA) supplemented with 2 mM glucose, 1mM pyruvate, and 2 mM glutamine, and cells were incubated for 1 h in the non-CO_2_ incubator. The OCR and ECAR were measured at 37 °C using Seahorse XF HS Mini Analyzer (Seahorse Bioscience, Billerica, MA, USA) and Agilent Seahorse XF Cell Mito Stress Test (Seahorse Bioscience). The OCR and ECAR values were measured after Oligomycin, FCCP, and Rotenone and Antimycin A injections at the time point specified in the producer’s protocol. Each experiment was conducted using three biological replications. Both OCR and ECAR values were normalized by µg of protein in each well and the data were analyzed by Agilent Seahorse Analytics (Seahorse Bioscience). 

### 2.11. Lactate Concentration

The lactate concentration was measured by Lactate Colorimetric Assay Kit II (Bio Vision, #K627). Spheroid and adherent ASCs and DFATs mediums were collected and cells were simultaneously homogenized on ice with 150 µL of cold Lactate Assay buffer and centrifuged with 10,000× *g* for 15 min at 4 °C to remove insoluble material. The cell supernatants and culture mediums were directly loaded on a 96-well plate (ThermoFisher Scientific), each at 25 µL of volume. The Lactate Standards were prepared and applied according to the manufacturer’s instructions. The plate was incubated for 30 min and the absorbance was measured at 450 nm by spectrometric plate reader FLUOstar OMEGA spectrophotometer (BMG LabTech). The lactate concentration was calculated according to the manufacturer protocol and normalized by 1 µg of total protein in each well.

### 2.12. Reactive Oxygen Species Production

The intracellular Reactive Oxygen Species (ROS) accumulation was measured with the use of CellROX™ Deep Red Flow Cytometry Assay Kit (C10491, Invitrogen) parallelly with the live/dead assessment using Sytox Blue Dead Stain dye (Invitrogen). ASCs-2D and DFATs-2D were seeded at a concentration of 2500 cells/ cm^2^ and ASCs-3D and DFATs-3D at a concentration of 0.1 × 10^6^ cells/mL. The adherent and spheroid ASCs and DFATs were cultured simultaneously and after 3 days of culture, the cells were collected and treated with 5µM CellROX™ Deep Red Reagent for 40 min at 37 °C in the dark. The Sytox Blue Dead Dye was added 15 min directly before ROS measurement. ASCs and DFATs intracellular Reactive Oxygen Species accumulation was measured in a standard culture medium using Flow Cytometry FACS Canto II and the results were analyzed with FACS Diva software (Beckton Dickinson) and FlowJo 10 (Beckton Dickinson).

### 2.13. Western Blot Analysis

Western blot analysis was performed for selected respiratory chain proteins (Total OxPhos Rodent WB Antibody Cocktail; 1:1000, ab458099, ThermoFisher Scientific), as previously described [47] and for the following proteins: phosphofructokinase-1 (PFK-1) (1:250, 55028-1-AP, Proteintech), phosphofructokinase-2 (PFK-2) (1:250, #13123, Cell Signaling) and glucose-6-phosphate dehydrogenase (G6PDH) (1:250, #12263, Cell Signaling). Spheroids and monolayer ASCs and DFATs were lysed in RIPA Lysis and Extraction Buffer (ThermoFisher Scientific) with the addition of Halt™ Protease and Phosphatase Inhibitor Cocktail (ThermoFisher Scientific). The cell extracts were separated by SDS-polyacrylamide gel electrophoresis with the use of any-kDa gels (Bio-Rad) with the amount of 40 µg of protein per line. Then, the cell extracts were electro-transferred onto a nitrocellulose membrane (Amersham, Sigma-Aldrich). In Western blot the following OxPhos complexes subunits were detected: NDUFB8 (complex I), CII-30 kDa (complex II); CIII-Core 2 subunit (complex III); C-IV-II subunit (complex IV); ATP synthase subunit alpha (complex V). All stripes were visualized by ECL™ Western Blotting Detection Reagents (Amersham™). The chemiluminescent signal was detected and quantified using the Fusion FX imaging system (Vilber Lourmat, Marne-la-Vallée, France). The band intensities of the proteins of interest were normalized to the total protein optic densities corresponding to the same lane visualized with Ponceau S and quantified using ImageJ software with a gel analyzer feature. 

### 2.14. ATP Content

The ATP concentrations experiment was based on a previously described method [48] with the use of a Colorimetric ATP Assay Kit (ab83355, Abcam, UK). ASCs-2D and DFATs-2D were seeded at 2500 cells/cm^2^ and ASCs-3D and DFATs-3D at 0.1 × 10^6^ cells/mL. After 3 days of culture, the cells were incubated in Krebs-Henseleit buffer (10 mM HEPES, 2 mMNaHCO_3_, 135 mM NaCl, 3.5 mM KCl, 0.5 mM NaH_2_PO_4_, 0.5 mM MgSO_4_, 1.5 mM CaCl_2_, 1 mM pyruvate, pH 7.4, Sigma Aldrich) with or without glucose (5.6 mM, Sigma-Aldrich), ATP synthase inhibitor: oligomycin (0.1 μg/mL, Sigma-Aldrich), glycolysis inhibitor: iodoacetate (1 mM, Sigma-Aldrich) or a mix solution containing oligomycin and iodoacetate for 30 min at 37 °C. After the incubation, the buffer was removed and the cells were collected and lysed in 150 µL of ATP Assay buffer. Then, the samples were centrifuged for 5 min at 4 °C at 13,000× *g* to remove insoluble material and loaded to a 96-well plate (ThermoFisher Scientific) at a volume of 50 µL in 3 technical replies. The plate was incubated at room temperature for 30 min and protected from light. The absorbance was measured at wavelength λ  =  570 nm by the FLUOstar OMEGA spectrophotometer (BMG LabTech). The experiment was conducted using four biological replications and the collected data were calculated according to the producer’s instructions. The data were presented as an ATP concentration (nmol) per 1 mg of total protein in each well. 

### 2.15. Statistical Analysis 

Raw data were analyzed by GraphPad Prism 7.0 software (GraphPad Software, San Diego, CA, USA) with a one-way analysis of variance (ANOVA), followed by Tukey’s post hoc test. The two-group experiments were analyzed by the t-Student test. The results are presented as a mean value of 3–5 independent biological replies. Statistically significant values were considered with the *p*-value < 0.05.

## 3. Results

### 3.1. ASCs and DFATs Characteristics

#### 3.1.1. Properties of Mesenchymal Stem Cells 

**Figure 2:**
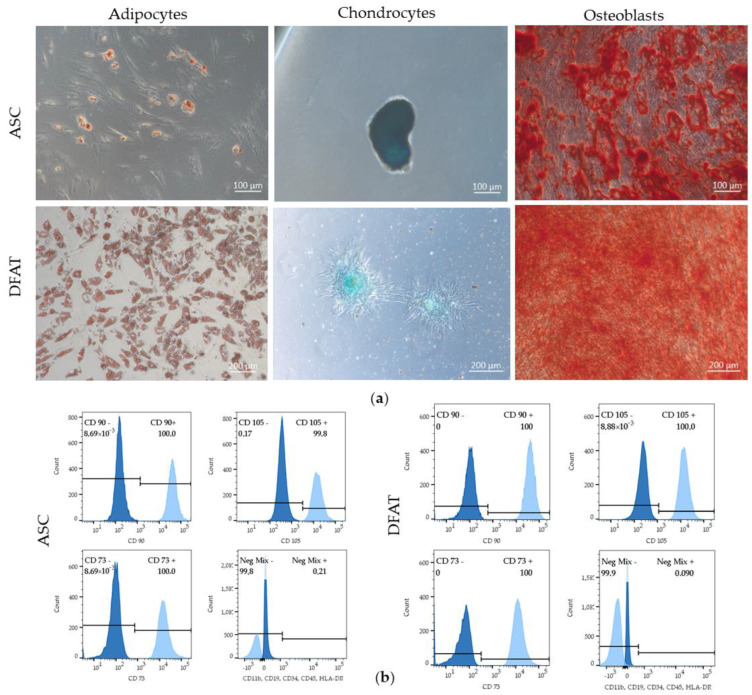
Properties of mesenchymal stem cells. (**a**) ASCs and DFATs differentiation into adipocytes, chondrocytes and osteoblasts. (**b**) Flow cytometry analysis of ASCs and DFATs; the presence of mesenchymal markers: CD73, CD90, CD105 and the absence of: CD34, CD11b, CD19, CD45 and HLA-DR at 2nd passage of culture.

**Figure 3:**
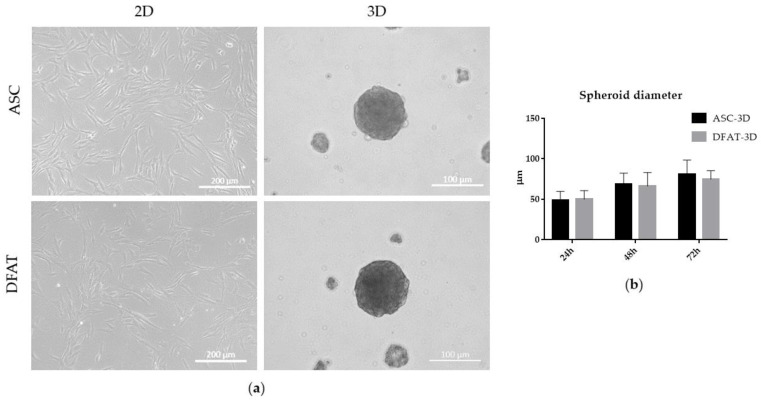
Assessment of ASCs and DFATs morphology cultured in 2D and 3D conditions. (**a**) Fibroblast-like (2D) monolayer ASCs and DFATs cultured on a standard plastic plate. Scale bar = 200 µm and three-dimensional (3D) spatial cultures of ASCs and DFATs created on the anti-adhesive surface. Scale bar = 100 µm; (**b**) Average spheroid diameter measured at 24, 48, 72, and 96 h of culture in ASCs-3D and DFATs-3D spheroids. The results are presented as mean values of 3 experiments ± SD.

Both populations: ASCs and DFATs met the stemness criteria, as they were tested positive for the presence of stemness markers: CD90, CD73, and CD105 and the absence of the markers: Cd11b, CD19, CD34, CD45, HLA-DR. ASCs and DFATs were also able to differentiate into adipocytes, chondrocytes and osteoblasts (Figure 2a,b), were able to adhere to the plastic surface and exhibited a fibroblast-like morphology (Figure 3a).

#### 3.1.2. Morphology and Spheroid Diameter

After being seeded on the anti-adhesive surface plates and after enriching their environment with bFGF and EGF, both ASCs and DFATs were able to spontaneously form three-dimensional spheroids floating in the culture medium (Figure 3a,b). The spheroids diameter was observed to increase from 49.0 µm ± 10.6 (ASC-3D) and 49.8 µm ± 10.7 (DFAT-3D) at 24 h of culture to 68.5 µm ± 13.7 (ASC-3D) and 66 µm ± 16.8 (DFAT-3D) after 48 h, and it finally reached maximal values of 80.4 µm ± 17.9 (ASCs-3D) and 74.7 µm ± 10.5 (DFATs-3D) diameters after 72 h in suspension (Figure 3b). After 96 h, a slow disintegration of both ASCs-3D and DFATs-3D populations was detected. The time of 72 h was considered the most optimal for the cell growth in spheroid cultures and it was chosen for further experiments. For the standard adhesive ASCs-2D and DFATs-2D cultures the 2nd passage was used in further experiments.

#### 3.1.3. Cell Viability

**Figure 4:**
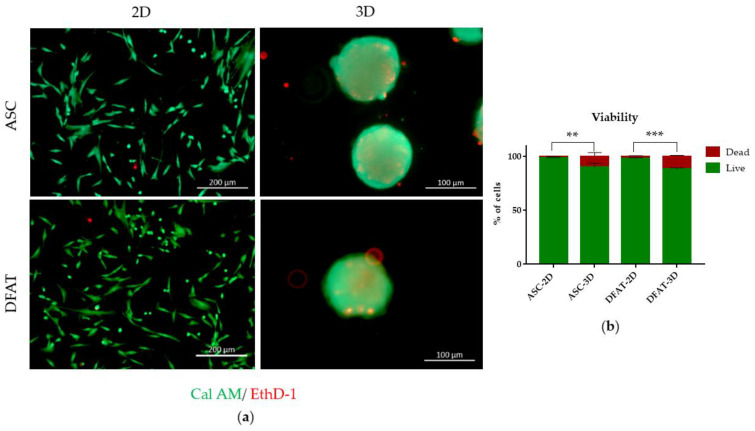
The viability of ASCs and DFATs cultured as a monolayer and as a spheroid. (**a**) Fluorescent microscope images of ASCs and DFATs population, living cells stained by green CalceinAM (CalAM), dead cells stained by red Ethidium homodimer-1 (EthD-1). Scale bar= 200 µm (2D) and 100 µm (3D); (**b**) The average percentage of dead and live cells in populations of ASCs and DFATs cultured in 2D and 3D conditions. The results are presented as mean values of 3 experiments ± SD, for ** *p* < 0.01, *** *p* < 0.0001.

The comparative analysis of ASCs and DFATs viability revealed that the majority of cells in both culture types were alive. In the monolayers, live CalAM-positive cells reached 98.6% ± 0.3 in ASCs-2D and 98% ± 0.7 in DFATs-2D. In 3D cultures, the percentage of alive cells was 90.1% ± 3.6 in ASCs-3D and 88.4% ± 1 in DFATs-3D. More dead cells were present in 3D culture (Figure 4a,b).

#### 3.1.4. Proliferation Potential 

**Figure 5:**
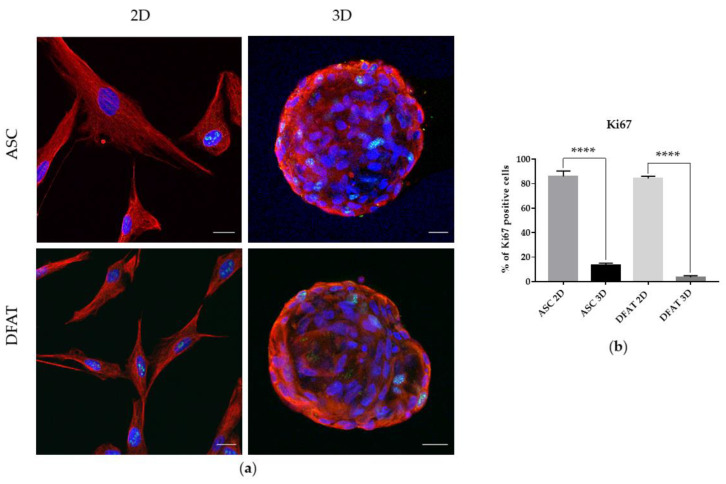
Comparative analysis of ASCs and DFATs proliferation abilities in adhesive and spatial culture. (**a**) The immunofluorescent visualization of cell proliferation potential with Anti-Ki67 (green). Anti-Fibronectin (red). Scale bar = 20 µm; (**b**) The percentage of Ki67 positive cells in the total population. The results are presented as mean values of 3 experiments ± SD, for **** *p* < 0.0001.

As shown by immunofluorescent staining, 3D culture of ASCs and DFATs significantly decreased their proliferation potential. The expression of the intra-nuclear Ki67 marker was reduced in the ASCs and DFATs spheroids in comparison to the monolayer culture, as the percentage of proliferating cells decreased from 86% ± 4.4 of Ki67 positive cells in ASCs-2D to 13.2% ± 1.8 in ASCs-3D and from 84.3% ± 1.9 in DFATs-2D to 3.7% ± 1.2 in DFATs-3D. (Figure 5 a,b). 

#### 3.1.5. Stemness-Related Transcriptional Factors (SRTF) Expression 

**Figure 6:**
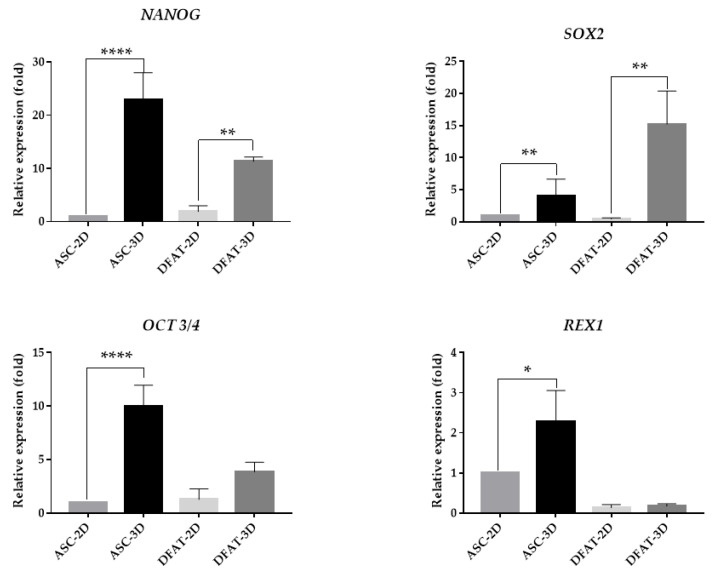
Relative expression fold values of pluripotency genes: NANOG, SOX2, OCT 3/4, and REX1 with β-ACT as a reference gene and ASCs-2D as the control group. The results are presented as mean values of 5 experiments ± SD, for * *p* < 0.05, ** *p* <0.01, **** *p* < 0.0001.

The qPCR analysis of SRTF genes showed that spatial culture conditions significantly increased the relative expression fold of NANOG, SOX2, OCT 3/4 and REX1 genes. In ASCs-3D culture, we observed almost 23-fold higher values of the NANOG gene expression: 22.9 ± 5.4-fold higher expression of SOX2 gene (4.1 ± 2.6), almost 10-fold higher OCT 3/4 gene expression (9.96 ± 2) and 2-fold higher values of REX1: 2.3 ± 0.8 gene expression compared to ASCs-2D, whose genes expression values were referred to as controls and represented 1 ± 0 relative expression fold. (Figure 6a). Similar tendencies were observed in DFATs-3D in which we recorded approximately 11-fold higher levels of NANOG: 11.3 ± 0.8 and a 15-fold increase of SOX2: 5.2 ± 5.2, a 3-fold higher expression of OCT 3/4: 3.8 ± 0.9, and 2-fold higher values of REX1 (2.4 ± 3.1) expression in comparison to the control group (Figure 6a).

### 3.2. Mitochondrial Content 

**Figure 7:**
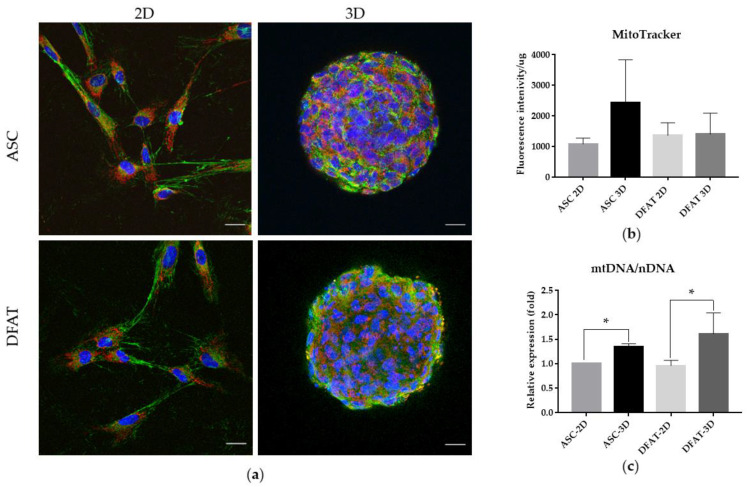
Comparative assessment of mitochondrial content in ASCs and DFATs cultured as a monolayer and spheroids (**a**) The immunocytochemical staining of mitochondria: Anti-mitochondria antibody (red) and Ani-Fibronectin antibody (green). Scale bar = 20 µm. (**b**) MitoTracker™ FM fluorescence intensity (**c**) The mtDNA/nDNA ratio. The results are presented as mean values of three experiments ± SD and are normalized per µg of protein, for * *p* < 0.05.

Immunofluorescent staining with an anti-mitochondrial antibody was performed to observe mitochondrial shape and arrangement in 2D and 3D cultures (Figure 7a). The mitochondrial content was further analyzed with MitoTracker Green ^TM^. We did not observe significant differences in the MitoTracker Green ^TM^ fluorescence intensity between ASC-3D and DFAT-3D spheroids in comparison to ASCs-2D and DFATs-2D (Figure 7b).

In order to determine the mitochondrial content more precisely, the mitochondrial DNA to nuclear DNA ratio (mtDNA/nDNA) was assessed. The qPCR analysis of mtDNA/nDNA showed that spheroid cultures significantly upregulated mtDNA content in both ASCs-3D and DFATs-3D. The values of mtDNA/nDNA ratio reached 1.3 ± 0.1 for ASCs-3D and 1.6 ± 0.4 for DFATs-3D and were elevated in comparison to the ASCs-2D whose mtDNA/nDNA value amounted to 1 ± 0 (Figure 7c). 

### 3.3. Metabolic Phenotype 

**Figure 8:**
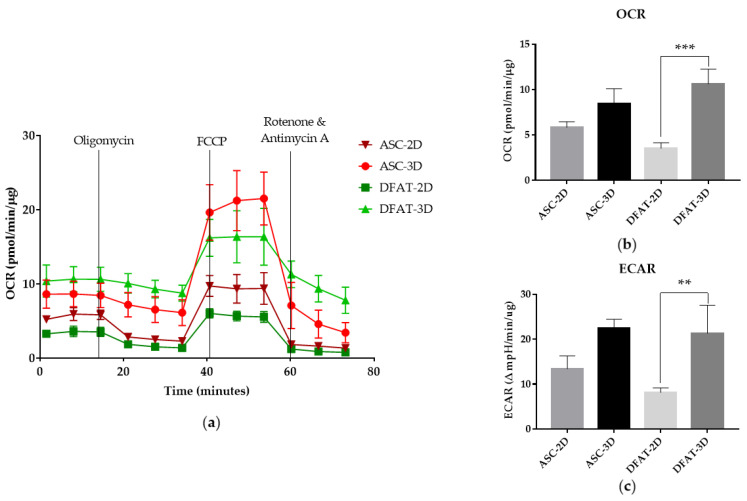
Metabolic phenotype evaluation by Seahorse XF HS Mini. (**a**) The kinetic graph of oxygen consumption rate (OCR) in time. (**b**) OCR mean values of basal respiration. (**c**) The extracellular acidification rate (ECAR) mean values of basal respiration. The results are presented as a mean value of 3 experiments ± SD and are normalized per µg of protein for ** *p* < 0.01, *** *p* < 0.001.

The metabolic phenotyping of the cells was carried out using the Seahorse XF HS Mini analyzer, which allows for a simultaneous measurement of two main metabolic pathways responsible for energy transduction for ADP phosphorylation in cells: glycolysis and mitochondrial respiration. The analysis of OCR- index of oxygen consumption rate, which is a direct determinant of mitochondrial respiration that indirectly measures the activity of the oxidative phosphorylation system, and ECAR-an index of extracellular acidification of the environment, which is directly related to the activity of the process of anaerobic glycolysis in cells, were carried out in living cells, in a non-destructive manner. Our analysis of energy metabolism showed that both 2D and 3D populations responded correctly to oligomycin, which inhibits ATP synthase activity, thereby reducing the oxygen consumption to a significantly lower level. Then, FCCP, protonophore, which completely abolishes mitochondrial membrane potential, was added to the cells. In the presence of this uncoupler, oxygen consumption reached the maximal rate because dissipation of the potential on the inner mitochondrial membrane caused a sharp increase in oxygen consumption. Finally, Rotenone and Antimycin A (inhibitors of complex I and III, respectively) were added to estimate the rate of respiratory chain-independent oxygen consumption. The course of the experiment was depicted by a kinetic plot of OCR over time (Figure 8a). 

The metabolic phenotype analysis revealed that the spheroid culture of cells exhibited a significant increase in mitochondrial respiration, as manifested by a higher basal OCR of three-dimensional cultures in comparison to monolayer cultured cells in both tested populations of ASCs and DFATs. 

The basal OCR was recorded at 8.4 ± 1.6 pmol/min/µg for ASCs-3D, while 5.8 ± 0.6 pmol/min/µg for ASCs-2D. Moreover, O_2_ consumption per minute was also significantly faster in DFATs-3D than in DFATs-2D, reaching 10.6 ± 1.6 pmol/min/µg in comparison to 3.5 ± 0.6 pmol/min/µg, respectively (Figure 8b). Moreover, conditions ECAR was more progressive in the ASC-3D spheroid culture than that observed in the cells grown in a monolayer ASCs-2D, 22.4 ± 2 mpH/min/ug vs. 13.4 ± 2.9 mpH/min/ug. A similar correlation was observed in the DFATs population, where the extracellular acidification rate was higher in DFATs-3D (21.3 ± 6.3 mpH/min/ug) than in DFATs-2D (8.14 ± 1 mpH/min/ug) (Figure 8c).

### 3.4. The Reactive Oxygen Species and Lactate Production 

**Figure 9:**
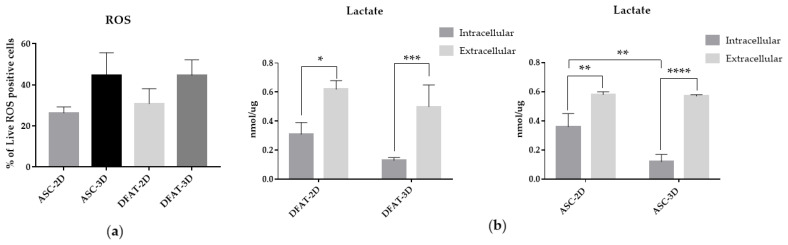
(**a**) The intracellular Reactive Oxygen Species (ROS) accumulation in live cells. (**b**) The intracellular and extracellular lactate production. The results are presented as mean values of 3-4 experiments ± SD and normalized per µg of protein, for * *p* < 0.05, ** *p* <0.01, *** *p* < 0.001, **** *p* < 0.0001.

The intracellular Reactive Oxygen Species analysis showed an increased ROS production in spheroids in comparison to monolayer cultures for both, ASCs and DFATs. ASCs-3D exhibited 44.5 % ± 11.1 of live cells with ROS accumulation and DFATs-3D: 44.6% ± 7.5, while ASCs-2D: 26.3% ± 2.9 and DFATs-2D: 30.8% ± 7.3 values of live cells with ROS accumulation (Figure 9a). 

Moreover, we showed that the 3D culture might significantly lower intracellular lactate production as compared to cells cultured as a monolayer. We found that ASCs-3Dproduced 0.12 nmol/µg ± 0.05 of Lactate, while DFATs-3D generated approximately 0.13 nmol/µg ± 0.02. In monolayers, the values were higher and up to 0.36 nmol/µg ± 0.09 in ASCs-2Dand 0.31 nmol/µg ± 0.08 in DFATs-2D. The differences in extracellular lactate concentration were not statistically significant and they ranged from approximately 0.6–0.5 nmol/ µg ± 0.1–0.15 nmol/ µg (Figure 9b).

### 3.5. Oxidative Phosphorylation Subunits Protein Levels 

**Figure 10:**
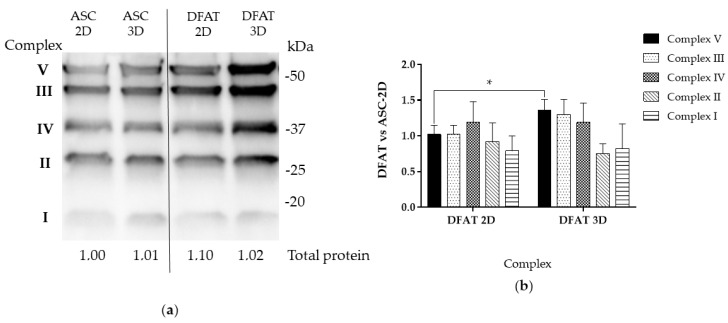
Assessment of protein levels of selected subunits of OXPHOS complexes. (**a**) Representative blot (**b**) Relative amounts of proteins counted in relation to ASC-2D. The results are presented as mean values of 3–5 biological replications and are normalized per µg of protein ± SD, for * *p* < 0.05.

The Western blot analysis of the selected subunits of oxidative phosphorylation complexes revealed no significant differences between the monolayer and spheroid culture of ASCs. However, three-dimensional conditions were observed to increase the level of particular OXPHOS proteins in the DFAT population. We observed a 1.36 ± 0.15 increase in complex V protein level in DFATs-3D compared to the reference group (ASCs-2D) (Figure 10 a,b). The relative protein levels of the subunits of complexes III and IV in DFATs-3D were also slightly elevated, however, with no statistical significance.

### 3.6. ATP Content

For ASCs, no significant differences in ATP content were observed between 2D and 3D cultures. The absolute amounts of ATP produced by untreated ASCs were significantly higher in the presence of glucose, reaching 19.0 ± 5.2 nmol/mg for 2D and 14.4 ± 3.8 nmol/mg for 3D, while 10.2 ± 1.4 nmol/mg for 2D and 9.11 ± 0.6 nmol/mg for 3D in the glucose-free medium. 

In addition, effects of oligomycin and iodoacetate on ATP content in 2D and 3D ASCs cultures were comparable. In the absence of glucose, a slight, statistically insignificant increase in ATP content was observed in both 2D and 3D cultures treated with iodoacetate. In glucose-free medium an incubation with oligomycin resulted in a minor drop in ADP phosphorylation in both types of ASCs culture. A slight reduction in ATP content was also observed for oligomycin-treated ASC in the presence of glucose. However, in the presence of glucose an iodoacetate reduced ATP content substantially in 2D and 3D, however, no statistical significance was recorded. Thus, the type of culture seems to have little effect on ASCs capacity for ATP biosynthesis. The ASCs seem to rely on both pathways of ATP formation-anaerobic glycolysis and oxidative phosphorylation- and the contribution of each of them may be dependent on the glucose availability.

The effects of various culture conditions (2D vs. 3D) were considerably more pronounced in DFATs populations and especially well-observed in the presence of glucose. In such conditions, ATP content in the DFATs spheres was almost twice as high as in the monolayer culture, reaching 26.6 ± 5.6 nmol/mg and 14.6 ± 2.4 nmol/mg, respectively. Moreover, in the presence of glucose, a significantly reduced ATP content was observed in DFATs spheres treated with iodoacetate (5.0 ± 1.66 nmol/mg). This effect was not observed for 2D DFATs culture, indicating more enhanced glycolysis in 3D DFATs in comparison to 2D culture. 

**Figure 11:**
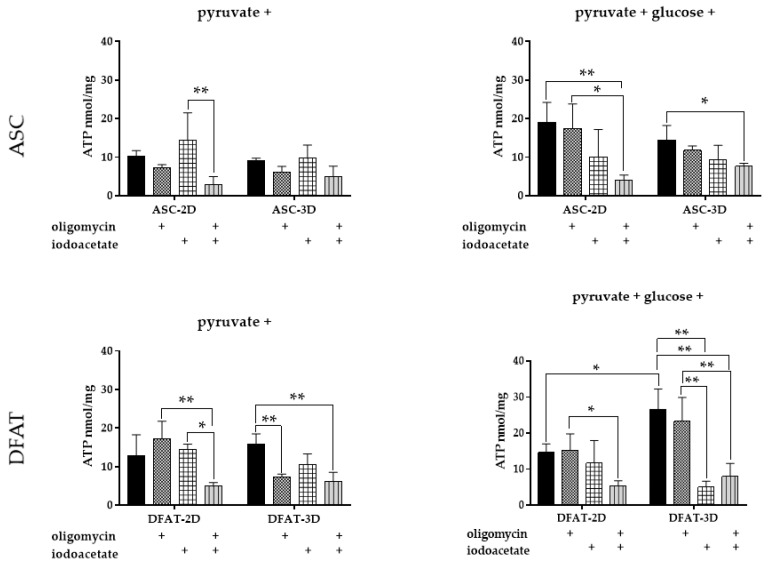
Comparative analysis of ATP content in ASC and DFAT cells treated with oligomycin and iodoacetate in the presence of glucose and in the glucose-free medium for 30 min. The results are presented as mean values of four experiments and are normalized per mg of protein ± SD, for * *p* < 0.05, ** *p* <0.01, “+”—presence of the inhibitor.

Further observation confirmed that an absolute ATP amount in glucose-free medium, when anaerobic glycolysis was limited, was very similar for 2D and 3D DFATs cultures, reaching 12.7 ± 5.6 nmol/mg and 15.9 ± 2.6 nmol/mg, respectively. Moreover, in a glucose-free medium an effect of iodoacetate was much more pronounced in 3D DFATs than in DFATs monolayer. Interestingly, a 2D DFATs culture seems to be insensitive to ATP biosynthesis modulators- oligomycin and iodoacetate- regardless of glucose availability (Figure 11a).

### 3.7. The Gene Expression of Main Glycolytic Enzymes

**Figure 12:**
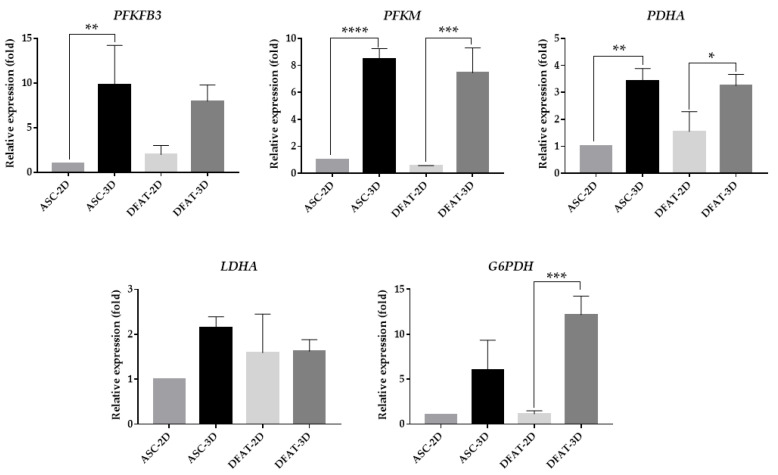
Relative expression of genes encoding for the main glycolytic enzymes in ASCs and DFATs cultured as a 2D monolayer and 3D spheroids. The results are presented as a mean value of 4 experiments ± SD, for * *p* < 0.05, ** *p* <0.01, *** *p* < 0.001, **** *p* < 0.0001.

The comparative qPCR assessment of the relative expression of main glycolytic enzyme genes showed that the 3D culture altered the transcript level of most of the considered genes and may influence glycolysis. We observed almost 10-fold higher values of *PFKFB3* expression in ASC-3D: 9.8 ± 4.4 and almost 8-fold higher values in DFATs-3D: 7.96 ± 1.8 in comparison to monolayer cultures. A similar tendency was found for *G6PDH*, the expression of which was 6-fold higher (6.0 ± 3.4) in ASCs-3D and approximately 12-fold higher (12.2 ± 2.1) in DFATs-3D. In the spheroid culture conditions, we also discovered a 3-fold higher expression of the *PDHA* gene in both tested populations ASCs-3D (3.4 ± 0.5) and DFATs-3D (3.3 ± 0.4). Spheroid culture conditions were also observed to influence *PFKM* gene expression. We detected an approximately 8-fold higher *PFKM* transcript content in ASCs-3D (8.5 ± 0.8) and DFATs-3D (7.5 ± 1.9) when compared to the monolayer control group. It was only the lactate dehydrogenase (*LDH)* relative expression that was comparable in the analyzed populations, and it was not affected by the culture form (Figure 12a).

### 3.8. The Protein Levels of Glycolytic Enzymes 

In our study, spheroid culture conditions were found to alter G6PDH protein level in ASCs. The Western blot analysis revealed higher protein levels of G6PDH in ASCs-3D, reaching 2.11 ± 0.8, in comparison to the ASCs-2D. For DFATs-3D, we also observed slightly higher values of G6PDH protein level but the difference was not statistically significant. 

**Figure 13:**
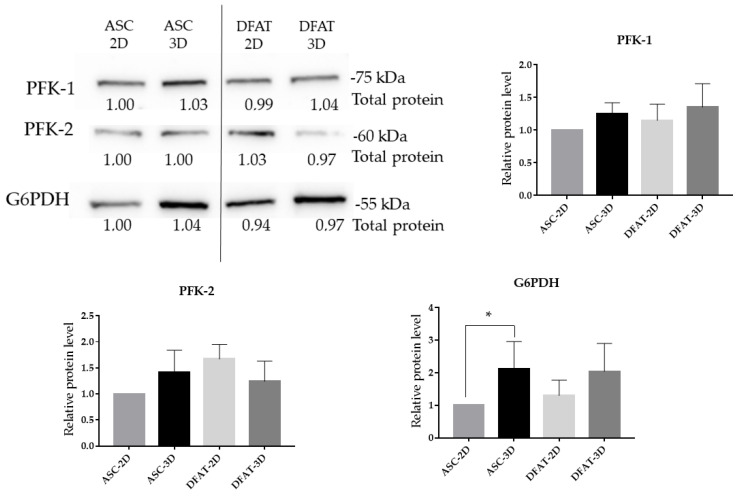
Relative protein levels of enzymes involved in glycolysis. The results are presented as a mean value of 3-4 biological replications and are normalized per µg of protein ± SD, for * *p* < 0.05.

Moreover, no significant differences in PFK-1 and PFK-2 protein levels were observed between cell populations and culture conditions (Figure 13a).

## 4. Discussion

The changes in the metabolism of the mesenchymal stem/stromal cells have already been investigated but some of the mechanisms still remain unclear. There are many doubts about the correlation between cell metabolism and culture conditions (2D vs. 3D in 5% O_2_), which may influence the cellular properties.

In the natural cell niche, under physioxic conditions, cells appear to be in a quiescent, primarily glycolytic, low-energy state and their proliferation rate is limited. However, when transferred to an artificial 2D environment with atmospheric oxygen cells reconfigure their metabolism to oxidative phosphorylation, such changes affect the properties of MSCs, e.g., leading to accelerated, excessive senescence and a decrease of cell plasticity [49,50].

In our study, we provoked the spontaneous formation of three-dimensional structures from ASCs and DFATs to mimic the natural cell niche environment. We observed that 3D form of culture limited the duration of cell proliferation to 72 h and then the proliferative potential decreased. Our observation corresponds to other authors’ general observations [9,51]. The differences between groups in the optimal 3D culture time for MSCs (from 4 to 10 days) [11] might result from the initial number of cells, the cell types and the adopted method [52,53]. However, the extended spheroid MSCs culture—unlike, e.g., embryonic stem cells—does not allow for a long-term cell cultivation. Some groups use for sphere creation different hydrogel scaffolds or magnetic sorting but such spheres more resemble aggregates and respond differently to environmental conditions [54,55].

Considering the proliferation capabilities, we observed that the spatial culture almost completely abolished MSCs proliferation potential. Reduced proliferation potential shown by a decrease of Ki67 expression or BrdU incorporation was also described previously [11,52,56]. Such a limitation of the proliferation rate might mimic the features of the quiescent cells in a niche or might precede cell death due to insufficient oxygen and nutrient penetration. What is more, live/dead staining revealed that in spheroids a larger number of dead cells is observed. This may also be caused by poor access to nutrients and limited oxygen flow through the inner structures of the spheroids, which has already been confirmed in previously published research [53,57]. Interestingly, despite a potentially reduced medium and oxygen enrichment in the spheroid core, their stemness-related transcription factors appeared to be elevated. Our data obtained in this study are consistent with some previous findings [9,12,53,58,59,60], as we demonstrated that the expression of stemness-related transcriptional factors (SRTF), such as *NANOG, SOX2, OCT 3/4*, and *REX1,* were upregulated in MSCs when cultured as spheroids. This response to three-dimensional culturing conditions may support stromal cell reprogramming to more stemness state or their dedifferentiation into developmentally younger cells, which positively correlates with increased secretion of growth factors and cytokines. Thus, presumably, the spheroid culture in which nutrients and oxygen access to the core is naturally reduced could potentially enhance its therapeutic properties. This may also explain the higher therapeutic properties of 3D-cultured cells [61].

As reported previously, a proliferative potential of the cells might be directly reflected by the activity of mitochondria [62], mitochondrial morphology, biogenesis, and even mitochondria transfer between cells [63]. All these changes may further influence MSCs’ senescence, apoptosis, self-renewal activity or differentiation capacities [28]. 

Here, we showed that spatial culture might result in increased mitochondrial content, as compared to the 2D conditions. By qPCR analysis, we demonstrated an increase in mtDNA content in spheroids, which has not been reported so far in the available literature. Moreover, three-dimensional conditions increased the oxygen consumption rate, which may also indicate more extended mitochondrial respiration in spheroids. However, we did not observe a significant difference in MitoTracker ™ fluorescence intensity.

Alterations in mitochondrial morphology and function induced by different culturing conditions were also previously reported. Liu et. al discovered a decrease in some mitochondrial abilities, such as electron transport and complex I activity together with an increased number of rounded mitochondria shapes after 3D culture, while Pennock et. al reported downregulation of electron transport chain-associated genes accompanied by enhanced presentation of shrunken, immature mitochondria in 3D-MSCs on transmission electron microscopy (TEM) micrographs.

It is also known that stem cell metabolism can adapt to prevailing environmental conditions as needed. For example, during differentiation, cell metabolism might be rearranged to be more aerobic. The increase in oxygen consumption rate and decreased extracellular acidification rate were previously reported for MSCs during osteogenic or adipogenic differentiation [64,65,66]. We found that our spheroid MSCs also exhibited some differences in metabolic phenotype. The Seahorse XF analysis showed that MSCs cultured as spheroids were characterized by a higher OCR, which presumably revealed an increased activity of oxidative phosphorylation. Moreover, the value of ECAR, which indicates elevated acidification of the extracellular environment caused by lactate production in anaerobic glycolysis and CO_2_ release from the TCA cycle, was also significantly increased in MSCs spheroids in comparison to monolayer-cultured cells, especially in DFATs. These data indicates that three-dimensional conditions might be more preferable for MSCs. Metabolic processes in spheroid cultured cells probably occur in a more physiological manner reflecting natural arrangements in the mesenchymal stromal cell niche. Contrary to our results, Son et al. showed that OCR in 3D cultures of dental pulp-derived mesenchymal cells decreased on day 72 of culture. However, the results are not normalized for the amount of cell protein and the data presented by the team are not statistically significant. Another group of researchers, Pennock et al., showed that the oxygen consumption rate presented by the MSCs spheroids decreased over time of culture (day 5. of culture) but the difference may result from the used method. 

Real-time studies based on the oxygen uptake were followed by Western blot analysis on mitochondrial respiratory complexes. We assessed the level of selected proteins of mitochondrial respiratory chain complexes, which has not been studied before. 

We showed that the levels of mitochondrial complex proteins necessary for the proper functioning of aerobic respiration, especially complex V, were elevated in DFATs spheroids, which may indicate that these cells are more predisposed to switch to an aerobic metabolism when assembled as spheroids.

Several other parameters characterizing cell metabolism were analyzed to further investigate the metabolism of spatially cultured MSCs. To recognize the preferable metabolic pathway of three-dimensional and monolayer cultured cells we measured ATP production in intact cells and also in the presence of oligomycin, or iodoacetate. In our study, we did not observe changes in metabolic preferences and ATP content in ASCs with regard to the type of culture, both in the presence of glucose and in a glucose-free conditions. In the presence of glucose, glycolysis is a major source of ATP, while in the absence of this substrate a contribution of OXPHOS to fulfill cellular energy demand is relatively higher. We observed that in DAFTS, in the presence of glucose, after temporary ATPase inhibition with oligomycin, the amount of ATP increases significantly, while after inhibition of glycolysis, the ATP values are very low.

This may indicate that DFATs spheroids largely base their energy metabolism and ATP synthesis on the process of anaerobic glycolysis. Consistently, some reports suggested that MSCs grown as aggregates might rely more heavily on anaerobic glycolysis [67]. However, the ability to use anaerobic glycolysis under oxygen availability conditions, known as the Warburg effect, and rapid proliferation are the hallmarks of cancer cell metabolism [68]. In MSCs spheroids, we probably observed a different use of anaerobic glycolysis, unrelated to the rate of proliferation, but further studies are needed to understand these mechanisms [69].

Another aspect related to metabolic reconfiguration can be closely bound to an intracellular ROS accumulation. ROS are a direct byproduct of oxidative respiration, and the accumulation of these cytotoxic compounds impairs MSCs survival and proliferation and leads to cell death by apoptosis or autophagy. The excessive release of ROS is considered to be detrimental to MSCs viability [70]. However, up-regulated ROS have also been discovered to be essential during differentiation in MSCs [71,72] and may be crucial to initiate proliferation in MSCs after quiescence exit [73]. In previous reports, we found conflicting information on the values of ROS production in spheroids. Some reports showed increased generation of ROS [9] while others recorded its decrease [74], depending on the experimental model and adopted experimental methods. Here, we demonstrated that MSCs cultured as spheroids exhibited higher values of intracellular ROS accumulation, which is consistent with our previous results and may be correlated with higher mitochondrial respiration. The change in the spatial arrangement of cells in spheroids may be the trigger for sudden stress and intracellular ROS shedding or potentially, it is the mitochondrial matrix activity itself that causes ROS to enter the cytoplasm, and this high level is beyond the capacity of the cell’s antioxidant system.

Our results led us to focus more specifically on anaerobic glycolysis and its auxiliary pathways, such as the pentose-phosphate pathway (PPP). In most cells, pyruvate formed in glycolysis can be converted by lactate dehydrogenase to lactate with NAD+ production or by pyruvate dehydrogenase (PDH) to acetyl-CoA, involved in the Tricarboxylic Acid Cycle (TCA) [33,75]. It has been discovered that in hypoxic conditions LDH activity is up-regulated, which is also directly associated with hypoxia-inducing factor-alpha (HIF-1α) activity [76]. As previously reported, aggregates culture resulted in an increased lactate concentration and indicated a lower glucose uptake by MSCs aggregates [9,57,58,60]. Our findings showed that LDH gene expression did not change significantly when MSCs were grown in spheroids, but intracellular lactate production seemed to be increased. In contrast, the gene expression of PDHA, which is a key enzyme linking glycolysis and mitochondrial respiration, was significantly upregulated. 

For glucose-6-phosphate dehydrogenase, which is essential in the pentose-phosphate pathway, higher expression was found in DFATs-3D, while ASCs-3D showed higher values of G6PDH proteins. We observed that the gene for the enzyme 6-phosphofructokinase, which catalyzes the formation of fructose-1,6-bisphosphate, was also more highly expressed in DFATs-3D and ASCs-3D than in 2D cultures, but we did not observe any significant differences in the protein level of this enzyme (PFK-1). In addition, we discovered a similar relationship between gene expression and protein level for PFKFB3 (also known as PFK-2), which is a bifunctional enzyme responsible for the regulation of glucose metabolism by generating or degrading fructose-2,6-biphosphate (F-2,6-BP), an allosteric activator of PFK-1. The PFKFB activity is up-regulated mainly by hypoxia [33] and expressed predominantly in embryonic and cancer cells [77]. We observed an increase in PFKFB3 gene expression in ASCs-3D but again no differences in the protein level of this enzyme were present there.

Increased expression of glycolysis genes that does not translate into higher amounts of the protein may result from the relatively short duration of the 3D culture. Perhaps more time is needed to observe changes in the protein levels, but the increased cell death in the spheres after 72 h of their culture is the limiting factor.

Nevertheless, the observed changes in the gene expression level may indicate important differences in the involvement of glycolysis pathways in the metabolism of 2D and 3D cells, which was also discovered previously [78].

To summarize, in our study, increased oxidative respiration and mitochondrial content, together with up-regulated expression of the glycolytic enzymes and extracellular acidification, showed that the cells grown in the form of three-dimensional spheres intensified their metabolism, enhancing both mitochondrial respiration and anaerobic glycolysis in comparison to the cells cultured in two-dimensional conditions. The enhanced activity of these metabolic pathways in 3D cultures may suggest that the maintenance of the dedifferentiation state and the expression of pluripotency genes is energy-consuming and that both pathways of ATP acquisition are needed. 

Our comparative metabolic analysis also revealed some differences between ASCs and DFATs populations. Statistically significant differences were discovered in the levels of OCR, ECAR, the amount of ATP or the protein level of the subunit of complex V, which were more clearly manifested by DFATs-3D compared to ASCs-3D. DFATs cultured in spheroids and in reduced oxygen concentrations are likely to adapt to environmental conditions better and to intensify their both aerobic and glycolytic metabolism compared to ASCs-3D. Therefore, it seems reasonable to use DFATs to construct 3D structures, and this suggests their potentially superior therapeutic properties in comparison to ASCs.

There are a number of hypotheses as to why metabolic changes may occur in three-dimensional spheroids. Previous reports showed, for instance, that the flow of nutrients deeper into the sphere was impeded but adequate oxygenation of each cell was also hampered. In 2017, Murphy et al. used a microsensor to determine whether a hypoxic core was generated inside the spheroid and their theory was confirmed. As the cells in the core of the sphere remained in extreme hypoxia, their metabolism needed to be reconfigured to glycolysis and its auxiliary pathways. High ECAR values and high glycolytic enzymes genes expression, observed in our research, would also confirm this hypothesis. It also means that the cells in the outer layers with access to higher oxygen concentrations were still equipped with a large number of active mitochondria. They made up for oxygen deficiency and used intensive oxidative phosphorylation for this purpose, as was evidenced by the elevated oxygen uptake or ROS accumulation that we demonstrated. We also have to take into consideration that the sphere itself consisted of a heterogeneous mix of cells, each at a different metabolic stage and different respiratory phenotype [79,80], thus the results that we observed in this study are a mean effect coming from the responses of cells in different layers of the spheroid.

Moreover, spheroids freely suspended in the medium is still not an ideal model of the interactions taking place in the niche. It should be remembered that cells in their natural environment form much more sophisticated structures that adhere strictly to one another in a specific way and coexist with other types of cells in their environment, which makes such a complex machinery work.

There is growing evidence that the change in the metabolic profile of the cell is programmed directly by pluripotency factors involved in the formation of primary pluripotent cells in their natural niche and that the metabolic change from OXPHOS to anaerobic glycolysis precedes the induction of pluripotency [81,82]. In the case of spherical MSCs cultures, we deal with a highly advanced process of reconfiguration of metabolism and an associated change in pluripotency capacity.

Our results clearly indicate that spatial conditions of cell culture and reduced oxygen tension may alter the metabolism of spheroids in both studied types of cells: ASCs-3D and DFATs-3D; however, this effect is much more pronounced in the latter. Moreover, it is reasonable to use DFATs due to their potentially superior therapeutic properties when compared to ASCs. Our work is a prelude to understanding how the metabolic profile of primitive MSCs naturally functions in the niche and what external factors could drive these cells in the therapeutically desirable direction. To further investigate the reorganization processes of glycolytic metabolism and mitochondrial respiration of spheroid-forming cells, a more profound analysis of these mechanisms would be needed.

## 5. Conclusions

Our study confirms that the culturing conditions, in particular the 2D and 3D form of culture in physiological, 5% oxygen concentration, affect the metabolism of ASCs and DFATs and might influence their therapeutic properties. Metabolic changes may also result from limited nutrient and oxygen penetration to the inner structures of the spheroids. The limited environmental conditions significantly affect the fate of cells.

Our study showed that cells grown as spheroids presented an enhanced mitochondrial and glycolytic metabolism. At the same time, they slowed down the proliferation rate and redirected their metabolism to increase the expression of stemness factors. An intensified metabolism might be associated with the increased demand for energy which is needed to maintain the expression of pluripotency genes and might be associated with the transition of cells from the state of proliferation to the state of dedifferentiation.

## Figures and Tables

**Table 1:** Primary antibodies used for immunocytochemistry analysis.

Antigen	Source	Isotype	Dilution	Manufacturer	Catalog Number
Fibronectin	Rabbit polyclonal	IgG	1:400	Sigma-Aldrich	F3648
Vimentin	Mouse monoclonal	IgG1	1:200	Dako	M0725
Mitochondrial surface	Mouse monoclonal	IgG	1:1000	Merck	MAB1273
Ki67	Rabbit polyclonal	IgG	1:200	Abcam	AB15580

**Table 2:** Secondary antibodies used for immunocytochemical analysis.

Antigen	Fluorochrome	Isotype	Dilution	Manufacturer	Catalog Number
Alexa Fluor Goat (anti-rabbit)	Alexa 488	IgG	1:1000	Life Technologies	A11034
Alexa Fluor Goat (anti-mouse)	Alexa 546	IgG1	1:1000	Life Technologies	A21123

**Table 3:** Primers sequences used with qPCR.

Gene	NCBI Reference Sequence	Product Size	Primer Sequence (5′ -> 3′)
***ACTB***	NM_001101.5	250 bp	F: CATGTACGTTGCTATCCAGGC R: CTCCTTAATGTCACGCACGAT
***NANOG***	NM_024865.4	103 bp	F: GAACCTCAGCTACAAACAGG R: CGTCACACCATTGCTATTCT
***SOX2***	NM_003106.4	93 bp	F: GTGGAAACTTTTGTCGGAGA R: TTATAATCCGGGTGCTCCTT
***OCT3/4***	NM_001285986.2	331 bp	F: CCTGAAGCAGAAGAGGATCACC R: AAAGCGGCAGATGGTCGTTTGG
***REX1***	NM_001304358.2	107 bp	F: GCTCCCTTGAATGTTCTTTG R: GCCTGTCATGTACTCAGAAT
***PFKFB3***	NM_001145443.3	124 bp	F: GCTTATGGCTGCCGTGTGGA R: GCGGGGTGACACTATTGCGT
***G6PDH***	NM_001282587.2	115 bp	F: GCCACAGAACTCGGGACCTT R: AAGCCTTGCGGTTCTGGTCT
***LDHA***	NM_001135239.2	117 bp	F: CTGGATTCAGCCCGATTCCG R: TCCACTCCATACAGGCACACT
***PDHA***	NM_000284.4	111 bp	F: CGTCTGTTGAGAGAGCGGCA R: ACCTTGTTGCCTCTCGGACG
***PFKM***	NM_000289.6	101 bp	F: ACTCTGCCCTGCATCGGATC R: ACAGTGGCGGCCCATTACTT

## Data Availability

Not applicable.

## Abbreviations

ASCs	Adipose Derived Stem/Stromal Cells
DFATs	Dedifferentiated Fat Cells
ECAR	Extracellular Acidification Rate
FCCP	Carbonyl cyanide-p-trifluoromethoxyphenylhydrazone
G6PD	Gene encoding: Glucose-6-Phosphate Dehydrogenase
LDHA	Gene encoding: Lactate Dehydrogenase
MSCs	Mesenchymal Stem/Stromal Cells
NANOG	Homebx Protein
OCR	Oxygen Consumption Rate
OCT3/4	Octamer-Binding Transcription factor
OXPHOS	Oxidative Phosphorylation
PDHA	Gene encoding: Pyruvate dehydrogenase
PFK-1	Phosphofructokinase 1
PFK-2	Phosphofructokinase 2
PFKFB3	Gene encoding: fructose-2,6-biphosphatase 3
PFKM	Gene encoding: 6-phosphofructokinase
REX1	Zinc Finger Protein 42 Homolog
RT	Room Temperature
SRTF	Stemness Related Transcriptional Factors
